# Praziquantel treatment of dogs for four consecutive years decreased the transmission of *Echinococcus intermedius* G7 to pigs in villages in Lithuania

**DOI:** 10.1016/j.fawpar.2019.e00043

**Published:** 2019-03-14

**Authors:** M. Šarkūnas, Ž. Vienažindienė, C.A. Alvarez Rojas, K. Radziulis, P. Deplazes

**Affiliations:** a Department of Veterinary Pathobiology, Veterinary Academy, Lithuanian University of Health Sciences, Tilžės str. 18, LT-47181 Kaunas, Lithuania; b Institute of Parasitology, Vetsuisse Faculty, University of Zürich, Winterthurerstrasse 266a, CH-8057 Zürich, Switzerland

**Keywords:** Cystic echinococcosis, Control, Praziquantel, *E. granulosus*, G7, *E. intermedius*

## Abstract

*Echinococcus granulosus* sensu *lato* comprises a number of recognized species which cause cystic echinococcosis (CE) in humans and intermediate hosts. These species have particular geographic distributions, with *E. granulosus* sensu stricto (genotypes G1/2/3 and micro variants) being most widely spread. In Lithuania, *E. intermedius* (G7) is known to be the only species circulating between pigs and dogs but is also infecting cattle and humans. In fact, recent reports showed a rise of the incidence to 1.13 human cases/100,000 inhabitants/year. Most of the pigs reared on the backyard farms in Lithuania are slaughtered on site during the cold season (October–April) and are used for own consumption. Therefore, in this study, we examined the impact on taeniid transmission of treating dogs with baits containing an oral formulation of praziquantel every two months during the pig slaughtering season in endemic villages in Lithuania. This study started in November 2006 and ended in January 2011; the first dog treatment was administered in February 2007. The results show that the prevalence of *E. intermedius*, *E*. *multilocularis* and *Taenia* spp. decreased significantly in treated dogs from the second year of the study when compared to untreated dogs. The treatment of dogs also had an impact on reducing the incidence of CE in fattener pigs from 17.6% (2006-2007) to 3.8% (2008; *P* < 0.05) and in sows from 26.9% (2006-2007) to 3.6% (2008), and eventually to zero in 2010 (P < 0.05) in fatteners and sows as compared to animals from “control” areas (30.7% and 63.7%, respectively). The results document a significant decrease in the transmission of *E. intermedius* (G7) after treatment of dogs with praziquantel in a relatively short time on farm level in Lithuania. Taeniid prevalence in dogs remained low in 2017 in the areas where anthelmintic intervention was performed until 2010 and, surprisingly, it was also strongly reduced in control areas. Reduction of taeniid transmission is likely associated with a decrease in the number of dogs in the villages as well as an overall decline in backyard pig farming after 2014 due to the outbreaks of African swine fever in Lithuania.

## Highlights

• The prevalence of *E. intermedius, E*. *multilocularis* and *Taenia* spp. decreased significantly in dogs treated strategically during the home slaughter season for pigs.
• Dog treatment also reduced the incidence and intensity of CE in fattener pigs and sows.
• Treatment should target all dogs, not just free-roaming dogs.

## 1 Introduction

Cystic echinococcosis (CE), caused by larval stages of the dog tapeworm *E. granulosus* sensu *lato* (s.l.), is an important zoonosis with worldwide distribution (Deplazes et al., 2017). The parasite is usually transmitted from the environment to humans by hand-mouth contact (after exposure in the contaminated environment or direct contact with faeces of definitive hosts). Hypothetically, transmission has also been linked to water or foodborne sources (vegetables/fruit) but any source attribution is uncertain (Alvarez Rojas et al., 2018). Nevertheless, the tapeworm is considered one of the most important foodborne parasites in Eastern Europe (Bouwknegt et al., 2018). *Echinococcus granulosus* s.l. comprises a number of species and genotypes with phenotypic differences in morphology and biology (host preference). In fact, genetic differences in mitochondrial genes are the basis for the genotype classification (G-system) published by Bowles et al. (1992) as the most widely used method for the characterization of this parasite complex. Subsequently, this nomenclature has been widely used for clustering different strains at species level. However, controversy still exists about the nomenclature of some species. For example, the genotypes G6/7/8/10 have been traditionally placed together and named *E. canadensis* (Nakao et al., 2013; Nakao et al., 2015). However, other authors have argued that the cluster G6/7 should be differentiated from G8/10 and be named *E. intermedius* (Lymbery et al., 2015; Lymbery, 2017; Thompson, 2017). The only variant of *E. granulosus* s.l. present in Lithuania is the G7 genotype (Bružinskaitė et al., 2009; Deplazes et al., 2017) to which in this article we refer to as *E. intermedius*.

The factors contributing to the transmission of *E. granulosus* s.l. are largely known but their individual contribution to parasite transmission can differ significantly in different endemic areas. Socio-cultural factors determine the livestock spectrum in an area (e.g. pig production) or influence parasite transmission based on traditional habits (Macpherson, 2005). For example, slaughtering activities linked to religious celebrations were shown to have a direct impact on transmission in Kosovo (Alishani et al., 2017). Extensive livestock husbandry, especially for sheep, and the socio-economic status of rural populations connected with home slaughtering activities facilitate the access of dogs to infected offal (Romig et al., 2017). In theory, interventions to stop or at least decrease parasite transmission in domestic animals should be feasible. These include preventing dogs from having access to offal, professional supervision of slaughtering practices, safe disposal of offal, deworming of dogs and public health education. Such measures contributed to the successful elimination of CE in Iceland (Beard, 1973; Craig et al., 2017). On the other hand, improved general slaughtering hygiene without any specific control measures was sufficient in large parts of Central and Western Europe to reduce bovine CE caused by *E. ortleppi* (G5) (Deplazes et al., 2017). However, in areas with extensive sheep production, further measures have to be applied to control *E. granulosus* s.s. (e.g. registration and regular deworming of dogs, public health education, construction of elimination ditches for the correct disposal of offal, etc). Based on previous control programmes, detailed protocols have been developed, providing recommendations for the control of the parasite in consecutive phases, which in total can take longer than 30 years (Craig et al., 2017). Only a handful of control programmes in the world have achieved the elimination status of *E. granulosus* s.s., all of them in island settings: New Zealand (Davidson, 2002), Tasmania (Beard et al., 2001) and as previously mentioned Iceland (Beard, 1973). Many other control programmes have been implemented worldwide, for example in Cyprus (Economides et al., 1998), Rio Negro and Neuquén provinces in Argentina (Larrieu and Zanini, 2012), Uruguay (Cabrera et al., 2002; Larrieu and Zanini, 2012), Magallanes region in Chile (Serra et al., 1993) and China (Heath et al., 2006). Although these programmes have not succeeded in the elimination of the infection, they have certainly contributed to preventing an unknown number of human infections (e.g. in Northern Cyprus). Therefore, the contribution of control programmes with only partial success in decreasing parasite transmission should not be ignored as their public health effects are difficult to quantify.

An increase in human alveolar echinococcosis and CE has been reported in Lithuania in the last decades, with case numbers of CE increasing from 13 cases per year (0.39/10^5^ inhabitants per year) in 2005 to 35 cases per year (1.11 cases/10^5^ inhabitants per year) in 2009. Since then it has remained at a comparable high level until 2013 (1.15 cases/10^5^ inhabitants per year) (Bružinskaitė et al., 2009; Marcinkutė et al., 2015). As previously mentioned, the only species/genotype isolated from pigs and humans in Lithuania is *E. intermedius* G7 (Bružinskaitė et al., 2009; Marcinkutė et al., 2015). Prior to these publications, data related to the “pig strain” (later determined as G7) of *E. granulosus* was scarce. Danilevičius (1964), publishing in the Lithuanian language reported that the parasite was circulating between pigs and dogs and that the transmission of the parasite was mostly related to the slaughtering of home-reared pigs at home during the cold season. Later on, it was confirmed that pigs from small farms showed a higher prevalence of CE (13.2%; 95% CI 10.6–16.1) as compared to those reared in more intensive systems (4.1%; 95% CI 0.8–11.5) (Bružinskaitė et al., 2009). Based on the local transmission of *E. intermedius* on small farms in Lithuania, we hypothesized that regular treatment of village dogs with praziquantel during the most intensive pig slaughtering period should have a strong impact on reducing the contamination of the environment with taeniid eggs and subsequently preventing parasite transmission and incidences of CE infections in pigs.

## 2 Materials and methods

### 2.1 Study design and animals

The trial was carried out between December 2006 and January 2011. Furthermore, in 2017, dogs of the same areas were screened for the presence of taeniid eggs in their faeces. In total, 11 villages were selected according to slaughterhouse records for CE in the endemic area in the southwest of Lithuania, located between 36.7 and 47.6 km from the border with Poland. Infected pigs were traced back to the backyard farm where they were reared, according to their ear tag number. The villages with the highest numbers of CE cases in pigs were assigned randomly to 2 areas: “treatment areas” with anthelmintic intervention in the dog populations and “control areas” without interventions. Six villages (Sasnava, Bitikai, Kantališkiai, Surgučiai, Purviniškės and Bebruliškė) were assigned to the treatment area, while six other villages (Aukštosios, Baraginė, Dženčialauka, Puskelniai, Taukaičiai and Smilgiai) from the same endemic area were assigned to the control area. A highway separated the treatment and control areas. The shortest distance between control and treatment areas was 1.65 km. The total number of dogs in the selected villages was estimated based on the average number of dogs per household. For this purpose, the number of dogs was counted in 436 households visited in 2006 and the average was 1.46 dogs per household. Based on this number, the total number of dogs was estimated to be 953 (540 in the control and 413 in the treatment areas) from 653 households in all selected villages (370 in control and 283 in treated areas). From the total dog population estimated in both groups, between 62.5 and 72% were sampled and examined for the presence of intestinal helminths once a year in December during the period 2006–2010. The majority of dogs were tested each year; however, some dogs had to be withdrawn from the study (animals died or were given away), while additional new dogs which were brought by the same owners were included in the study. Most dogs were local crossbreeds, between 1 and 10 years of age with an average weight of 16 kg (8–50 kg). All dogs were included in the study with the consent of the owners. The owners of control and treated dogs were not blinded during the study. In 2017, 6 years after the end of the intervention, faeces of dogs from the same control and treatment areas were sampled to document the situation and assess the sustainability of the control efforts. The average number of dogs/household was 0.6, yielding a calculated number of 222 dogs in the control and 170 in the treatment areas. From these dog populations, 83.8% of dogs from the control areas and 87.2% from the treatment areas were sampled and examined.

### 2.2 Treatment of dogs

The dogs included into the treatment area were given baits containing praziquantel (Impfstoffwerk Dessau Tornau GmbH, Bayer AG, Germany) at a dose of 5 mg/kg body weight. The baits were offered to the dogs and were taken voluntarily by them. A team member supervised that the bait was actually swallowed by each dog. The treatment was given 4 times per year (in February, April, October and December) covering the time when most home-slaughtering of pigs are expected to occur (September–April) between 2007 and 2010. The two months interval between treatments was based on the prepatent period of *E. intermedius* of around two months (Danilevičius, 1964). The dogs in the control area did not receive treatment with praziquantel; however, their health status was assessed during the study. At the end of the intervention period, all dogs from the control and treatment areas were treated with praziquantel during the last sampling in December 2010.

### 2.3 Collection and examination of dog faecal samples

The faecal samples were collected every year during the treatment visit in December from all dogs included in the study. The faeces were collected individually from the ground. The dogs were classified as chained dogs, dogs in pens, or unrestrained dogs; in the last case defecation places were pinpointed by the owners. In total, between 218 and 394 dogs were sampled and examined each year in the period of 2006–2010 while faeces from 334 dogs were examined in 2017. Faecal samples were frozen at −80 °C for 5 days to inactivate taeniid eggs and subsequently stored at −20 °C until further use. The efficacy of the treatment strategy in dogs was measured by estimating the prevalence of patent taeniid infections, followed by DNA analyses for genus/species determination. Individual faecal samples were homogenised with a spatula and a subsample of 2.5 g was placed into a 12 ml tube, diluted with water (1:4) and centrifuged at 1600*g* for 10 min. The sediment (about 2 ml) was resuspended with 6 ml of ZnCl_2_ solution (density: 1.45) and vortexed vigorously. Tubes were filled with the same ZnCl_2_ solution and centrifuged for 30 min at 400*g*. The supernatant containing the eggs was passed through sieves of different sizes (100, 50 and 21 μm); the taeniid eggs (if present) were retained in the 21 μm mesh (Mathis et al., 1996). DNA extraction of eggs was performed as described previously (Štefanić et al., 2004). DNA was used as a template for a multiplex PCR according to Trachsel et al. (2007) for the simultaneous detection of *E. granulosus* s.l. (including *E. intermedius* G7), *E. multilocularis* and *Taenia* spp.

### 2.4 Collection and examination of pig viscera

The viscera (liver, lungs, kidney and spleen) of pigs raised in treatment and control areas were examined for the presence of CE. The collection of pig samples was performed in local abattoir and through the local veterinarian in case of home slaughtered pigs during the whole year in the period between November 2006 and December 2010. All pigs examined during the study were grouped by age into two categories: fatteners (up to 9 months of age; *n* = 758) and sows (over 10 months of age; *n* = 930). Of those, 350 fatteners and 617 sows were raised in control areas, while 408 fatteners and 313 sows were raised in treatment areas. The presence of cysts of *E. intermedius* was investigated in the liver. The infected livers were frozen at −20° C and later examined. *Taenia hydatigena* cysticerci were identified morphologically during post slaughter examination and not collected for this study. Livers were cut into 2 cm slices, all visible cysts were excised, opened and morphologically examined. All cysts were categorised into three groups according to morphological appearance i.e. developing cysts (surrounded by an intact laminated layer containing fluid without protoscoleces), fertile cysts (containing fluid and protoscoleces) and degenerating cysts (necrotic fibrotic lesions with a fragmented laminated layer without cyst fluid with or without protoscoleces). The occurrence of different groups of cysts in slaughtered pigs was recorded, and the annual incidence and also the intensity (the number of cysts per infected animal) of each group of cysts were calculated for pigs from control and treated areas. Samples with multiple cysts were considered positive for *E. intermedius* if at least one typical cyst was identified. DNA isolations from morphologically unidentifiable lesions (*n* = 5) were performed using a commercial kit (Qiamp DNA mini kit, Qiagen, Hilden, Germany) according to the manufacturer's instruction. PCR with these samples was done according to Bowles et al. (1992).

### 2.5 Questionnaire

A set of questions were asked to 619 household farmers (356 in control and 263 in treatment areas). The information included age, gender, name and brief physical description of the dogs; their feeding habits: offal (raw organs or meat) or kitchen leftover (boiled meat, porridge, vegetables, soups etc.) or commercial food; anthelmintic treatment (yes/no) and whether they were kept indoor, in pens, chained or unrestrained. The farmers were also asked for information on the timing of home slaughter of pigs.

### 2.6 Statistical analysis and other considerations

The exact binomial confidence intervals for *E. intermedius* infection between fatteners and sows in control areas and areas with anthelmintic intervention, the annual prevalence of *E. intermedius*, *E. multilocularis* and *Taenia* spp. in control and treated dogs were calculated using Interactive Statistical Calculation Pages (Pezzullo, 2009). The differences in excretion of *E. intermedius*, *E. multilocularis* and *Taenia* spp. eggs with dog faeces, the incidence of CE as well as the incidence of various groups of cysts in fatteners and sows in treatment and control areas, and the questionnaire results were analysed using Fisher‘s Exact test. The average number of cysts per infected pig in control and areas with the intervention was compared using two-sided Student *t*-test (Graph Pad Prism, version 4.0). A *p*-value ≤0.05 was considered statistically significant.

## Ethics statement

The study was conducted in compliance with the Lithuanian Animal Welfare Regulations (No B1-866, 2012; No XI-2271, 2012). The import and use of the oral formulation of praziquantel for the control experiment were in accordance with the permission No. S-475 issued by the Inspection of Veterinary Preparations, Lithuanian State Food and Veterinary Service.

## 3 Results

### 3.1 Infection of dogs

**Fig. 1:**
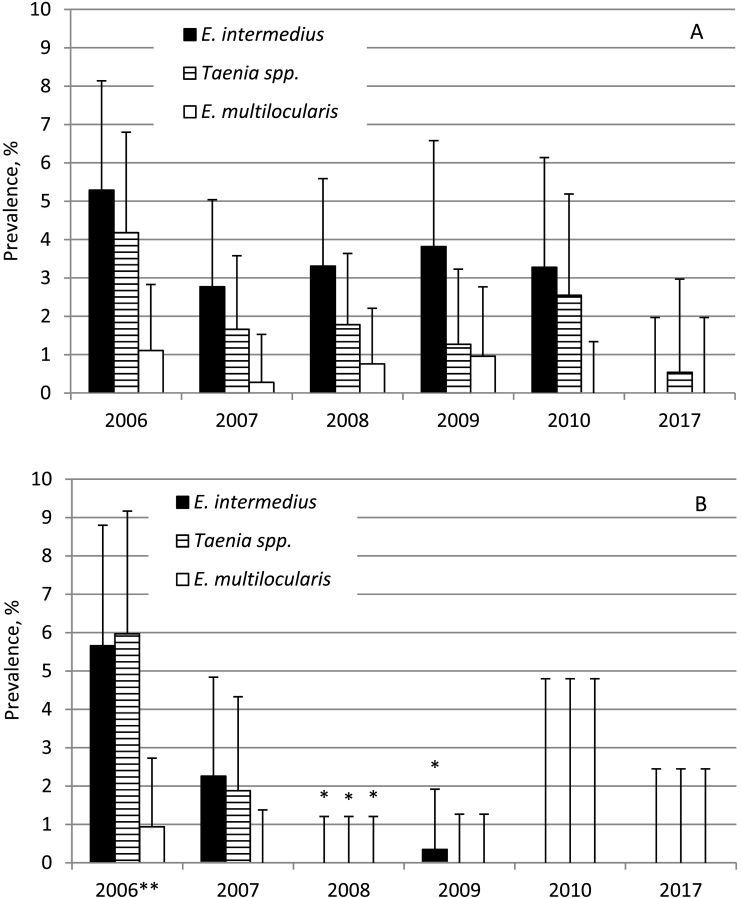
The prevalence of *E. intermedius, Taenia* spp. and *E. multilocularis* in control (A) and praziquantel treated (B) dogs examined in December of each year. Results are based on taeniid egg isolation and molecular identification (Trachsel et al., 2007). All dogs were treated with praziquantel at the last sampling in 2010, thus the data for 2017 represent comparable populations of dogs. Vertical bars represent 95% confidence interval. * significant reduction as compared to the control group in the same year (*p* < 0.05); ** before the first treatment.

The dogs from the control areas showed prevalences of *E. intermedius* between 2.8% and 5.3% (without significant differences) in the period 2006–2010 (Fig. 1A). Meanwhile, in dogs of the treatment areas the highest prevalence of *E. intermedius* (5.7%) was observed at the beginning of the study in 2006 before the first treatment was initiated (Fig. 1B). Significantly fewer dogs from the treatment areas excreted *E. intermedius* eggs in 2008 (0.0%; 95% CI 0.0–1.2; *P* < 0.05) and 2009 (0.4%; 95% CI 0.0–1.9; P < 0.05), while in 2010 (0.0%; 95% CI 0.0–4.8) the difference was not significant (*P* > 0.05) as compared to the control areas.

The excretion of *Taenia* spp. eggs ranged between 1.3% (95% CI 0.4–3.2) and 4.2% (95% CI 2.4–6.8) during the study in dogs of the control areas (Fig. 1A). Meanwhile, in dogs of the treatment areas (Fig. 1B), the prevalence of *Taenia* spp. decreased from 6.0% (95% CI 3.6–9.2) in 2006 to 1.9% (95% CI 0.6–4.3) in 2007. However, this difference was not significant (P > 0.05) as compared to the control areas. Although none of the dogs of the treatment areas was excreting *Taenia* eggs in their faeces in the period 2008–2010, the difference was only significant (*P* < 0.05) in 2008 when compared to the control areas.

The prevalence of *E. multilocularis* varied between 0.0% (95% CI 0.0–1.3) and 1.1% (95% CI 0.3–2.8) during the study, without significant variations in dogs of the control areas (Fig. 1A). In 2006, the prevalence in the treatment areas (0.9%; 95% CI 0.2–2.7) was comparable to those in the control areas (1.1%; 95% CI 0.3–2.8). However, in the following years, no *E. multilocularis* infections were detected in dogs of the treatment areas (Fig. 1B). In the control areas, in 2017 neither *E. intermedius* nor *E*. *multilocularis* was detected and only a low proportion of the samples was positive for *Taenia* spp. (Fig. 1a), while no taeniids were present in dog faeces from the treatment areas (Fig. 1B).

### 3.2 Cystic echinococcosis in pigs

**Fig. 2:**
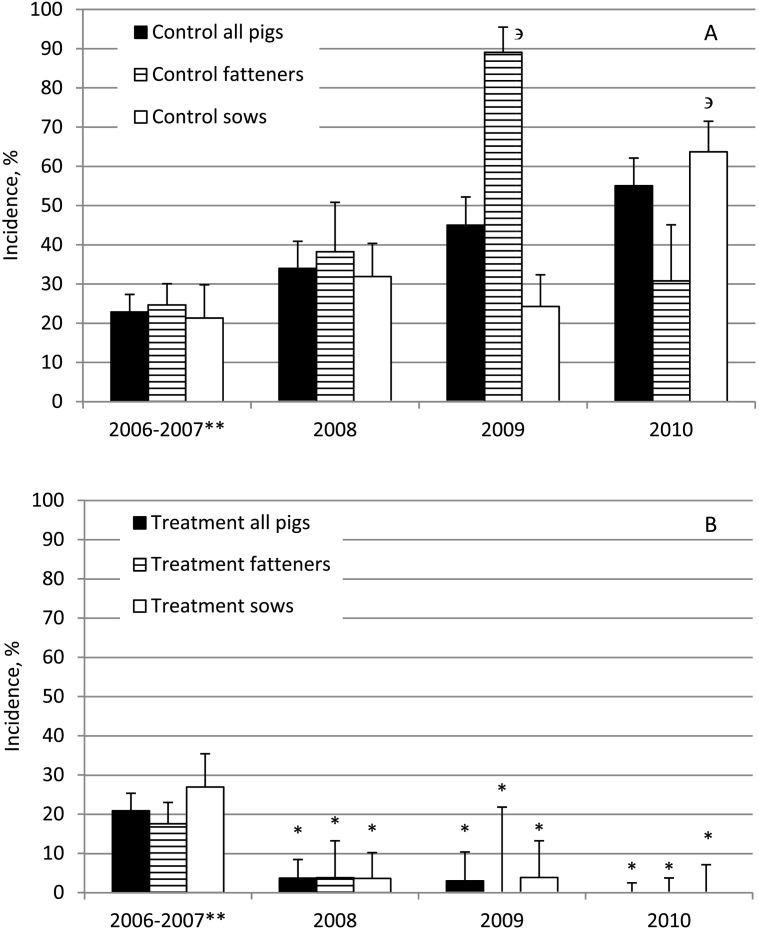
The annual incidence of cystic echinococcosis in all pigs investigated, in fatteners and in sows from control areas (A) and areas with anthelmintic intervention starting in December 2006 (B) according to post-mortem examination of carcasses from pigs slaughtered during the whole year. *significant reduction as compared to respective pigs from control areas in the same year (p < 0.05) or other group of pigs in the same year ^∋^ (p < 0.05); ** data collected in period between November 2006 and December 2007.

Due to the low number of pig samples in 2006, data from this year is presented together with data from 2007. The incidence of CE in pigs from control areas (Fig. 2A) ranged between 22.9% (95% CI 18.6–27.5) to 55.1% (95% CI 47.8–62.1) during the study. The annual incidence in fatteners and sows in this group was comparable in the period of 2006–2008. However, in 2009, the incidence in fatteners (89.1%; 95% CI 78.8–95.5) and in 2010 in sows (63.7%; 95% CI 55.3–71.5) increased significantly (*P* < 0.05) compared to those of sows (24.3; 95% CI 17.3–32.4) and fatteners (30.8%; 95% CI 18.7–45.1), respectively, in the same years.

The incidence of CE in pigs decreased significantly (P < 0.05) in 2008 compared to those in control areas and remained low during the rest of the study in the villages with anthelmintic intervention (Fig. 2B). In 2008, the incidence in fatteners (3.9%; 95% CI 0.5–13.2; P < 0.05) and sows (3.6%; 95% CI 0.8–10.2) became significantly lower as compared to those in the control areas (38.2% 95% CI 26.7–50.8 and 31.9%; 95% CI 24.2–40.4, respectively). Furthermore, the incidence of CE decreased to 0% (95% CI 0.0–21.8) in fatteners in 2009 and in both age groups in 2010 and remained significantly lower (*P* < 0.05) when compared to control areas.

### 3.3 Development of metacestodes

During the study, 737 developing cysts (mean size 11.4 ± 10.6 mm; 95% CI 10.7–12.2) from 141 pigs, 385 fertile cysts (mean size 29.0 ± 23.2 mm; 95% CI 26.7–31.3) from 128 pigs and 374 degenerated cysts (mean size 11.2 ± 9.6 mm; 95% CI 10.2–12.2) from 99 pigs were found. From 215 infected pigs, 76 (35.4%; 95% CI 29.0–42.1) harboured one group of cysts, 94 (43.7%; 95% CI 37.0–50.6) two groups of cysts while 45 (20.9%; 95% CI 15.7–27.0) pigs harboured three groups of cysts.

**Fig. 3:**
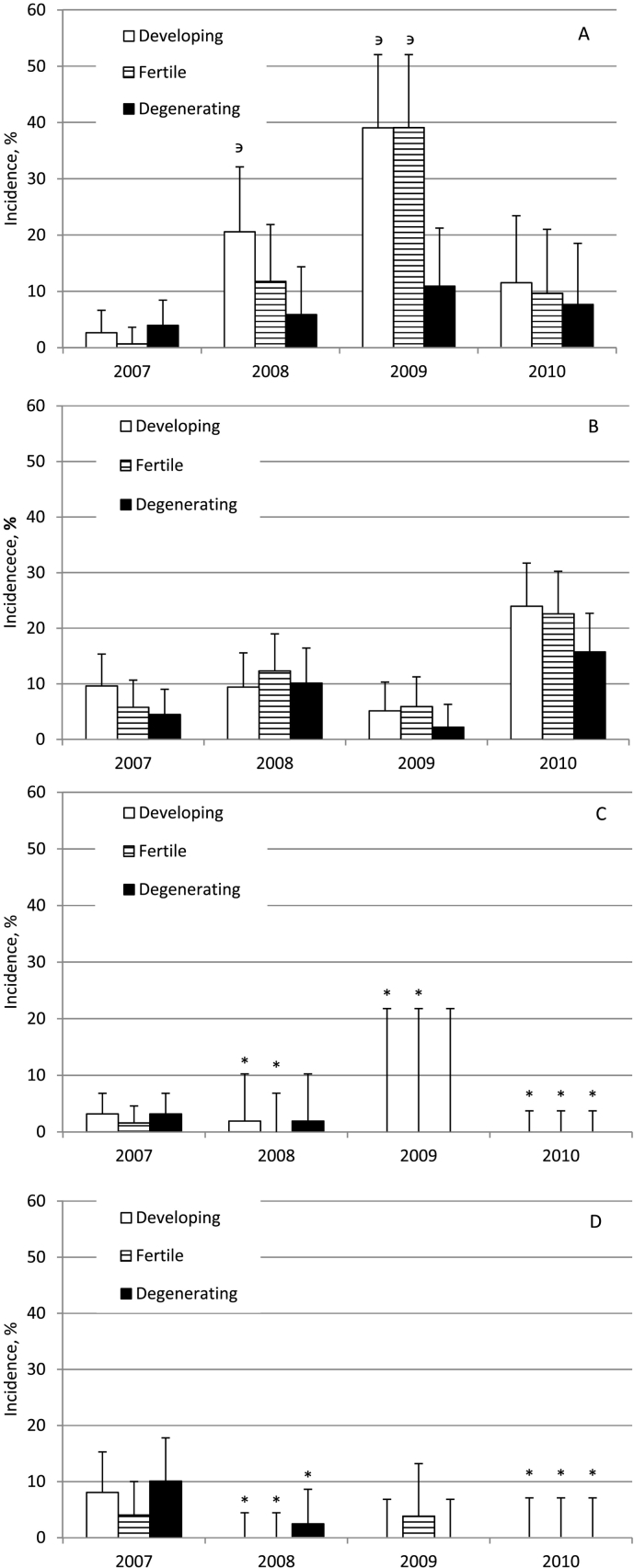
The annual incidence of developing, fertile or degenerating *E. intermedius* cysts in fatteners (A) and sows (B) from control areas and in fatteners (C) and sows (D) from areas with anthelmintic intervention. Vertical bars represent 95% confidence interval. * significant difference as compared to pigs from control areas in the same year (p < 0.05) or degenerating cysts in the same group within the same year ^∋^ (p < 0.05).

The results from the morphological analysis of cysts (Fig. 3) show that the incidence of developing, fertile and degenerating cysts isolated from pigs of the control areas was not constant in most of the years. In fatteners (Fig. 3A), the incidence of developing cysts (20.6% in 2008; 95% CI 11.7–32.1 and 39.1% in 2009; 95% CI 27.1–52.1) and fertile cysts (39.1% in 2009; 95% CI 27.1–52.1) was significantly higher (*P* < 0.05) than those of degenerating cysts (5.9% in 2008; 95% CI 1.6–14.4 and 10.9% in 2009; 95% CI 4.5–21.3) in the same years within the same group. Meanwhile, in sows (Fig. 3B) the incidence of developing, fertile and degenerating cysts was comparable in all years.

In the beginning of the study (2007) the incidence of developing, fertile and degenerating cysts was comparable (*P* > 0.05) in both areas (Fig. 3). Starting from 2008 the incidence of developing and fertile cysts were significantly reduced (*P* < 0.05) in fatteners (2008, 2009, 2010) (Fig. 3C) and in all groups of cysts in sows (2008, 2010) (Fig. 3D) in areas with anthelmintic intervention as compared to those of control areas.

In beginning of study (2007) the developing cysts (Table 1) were most frequently detected (*P* < 0.05) in pigs from control areas (54.0%) and from the areas with intervention (67.7%) as compared to those of other groups of cysts within the same area. In the period of 2008–2010, however, the average number of developing cysts decreased significantly (9.1%; P < 0.05) in pigs from the areas with intervention as compared to control areas (41.9%) in the same period. Additionally, the average number of cysts (not presented in figures) per infected pig decreased significantly (P < 0.05) in the areas of intervention (1.7 ± 1.2; 95% CI 0.4–2.9) in the period of 2008–2010 as compared to pigs from the control areas (3.5 ± 6.9; 95% CI 2.6–4.3) during the same period.

**Table 1:** The incidence of developing, fertile or degenerating *E. intermedius* cysts in pigs in 2007 (beginning of study) and 2008–2010 (during the intervention) from control areas and areas with anthelmintic intervention. * significant difference as compared to other group of cysts in the same period within the same area; ^϶^ significant difference as compared to the incidence of respective group of cysts in pigs from control areas in the same period (p < 0.05).

	The number of cysts in pigs in control areas			The number of cysts in pigs in the areas with intervention		
	Developing	Fertile	Degenerating	Developing	Fertile	Degenerating
2007						
N per category/N per year	74/137	24/137	39/137	256/378	32/378	90/378
Proportion of cysts, %; (95% CI)	54.0%* (45.3–62.6)	17.5% (11.6–24.9)	28.5% (21.1–36.8)	67.7%* (62.7–72.4)	8.5% (5.9–11.7)	23.8% (19.6–28.4)
2008–2010						
N per category/N per period	406/970	324/970	240/970	1/11	5/11	5/11
Proportion of cysts, %; (95% CI)	41.9%* (38.7–45.0)	33.4% (30.4–36.5)	24.7% (21.1–27.6)	9.1%^϶^ (0.2–41.3)	45.4% (16.7–76.6)	45.4% (16.7–76.6)

### 3.4 Questionnaire results

The results from the questionnaire showed that the majority (P < 0.05) of dogs (69.1%; 95% CI 66.4–71.7) were kept chained as compared to those kept in pens (15.8%; 95% CI 13.8–18.0), unrestrained (11.2%; 95% CI 9.5–13.1) or indoor (3.8%; 95% CI 2.8–5.1). This distribution was comparable in the control and treatment areas. Out of the 1220 dogs included in the study (2006–2010), only 341 (28%; 95% CI 25.4–30.6) received an anthelmintic treatment at least once before the study while the remaining 879 dogs (72%; 95% CI 69.4–74.6; P < 0.05) did not receive a treatment before the start of the study. The average incidence of *E. intermedius* was comparable (*P* > 0.05) in chained dogs (7.5%; 95% CI 5.2310.2), dogs kept indoor (6.7%; 95% CI 0.8–22.1), in pens (5.5%; 95% CI 1.8–12.4) and unrestrained (4.1%; 95% CI 0.9–11.5) control dogs. Similarly, the average incidence of *Taenia* spp. was comparably (P > 0.05) low in chained dogs (5.2%; 95% CI 3.4–7.6) when compared to dogs kept in pens (4.4%; 95% CI 1.2–10.9) or indoor (3.3%; 95% CI 0.1–17.2) dogs. Unrestrained control dogs were most frequently (P < 0.05) infected with *E. multilocularis* (average 5-year incidence 5.4%; 95% CI 1.5–13.4) when compared to those kept in pens (0%; 95% CI 0–4.0) or chained (1.2%; 95% CI 0.5–2.7).

## 4 Discussion

Praziquantel was developed in the 1970s proving to be an effective anthelminthic against a broad spectrum of parasitic trematodes and cestodes (Andrews et al., 1983). Since then, it has been used for mass treatment of dogs against intestinal taeniid infections, especially against *E. granulosus* s.l., becoming one of the key tools in >18 control programmes aiming to reduce human CE incidence worldwide (Craig et al., 2017). In our study in Lithuania, we used baits containing praziquantel, a formulation used in field *E. multilocularis* control experiments for deworming of red foxes (Hegglin and Deplazes, 2013). The baits were well accepted by the farm dogs without the time-consuming individual dosing when dogs are obliged to swallow the praziquantel tablets. The dogs were treated in two-months intervals based on experimental data from Danilevičius (1964). In these experimental infections with protoscoleces of pig origin, the average pre-patent period varied between 59.6 and 61 days in six pups (2.5–3 months) and six adult dogs (2–6 years), respectively.

Most of the transmission of *E. intermedius* from pigs to dogs takes place during the main slaughtering season in Lithuania, between October and May (Marcinkutė et al., 2015). The timing for the microscopical examination of dog faeces in December (two months after the last treatment) was chosen to evaluate the efficiency of the treatment strategy in comparison with the control areas. However, this monitoring did not allow detecting infections occurring from late spring to early autumn. Therefore, additional examination of dogs at the end of the summer season before the first treatment could be considered in the monitoring of a similar future control programme, especially if there is no seasonality in the transmission of *E. intermedius*.

The high specificity and sufficient sensitivity (94%; Mathis et al., 1996) of the sieving/PCR diagnostic strategy allowed the proper identification of Taeniidae eggs in this study. However, due to prepatent infections, sporadic segment or worm shedding and the relative low fecundity of *Echinococcus spp.,* the prevalence of intestinal infections might have been underestimated before and after the intervention in this study. Such inconsistency in detection of eggs was shown in the study on comparative copro-diagnosis in foxes experimentally infected with *E. multilocularis* (Al-Sabi' et al., 2007). During the low patent period (71–90 dpi) *E. multilocularis* eggs were detected in 23 out of 30 (77%) infected foxes while the highest (100%) detection of eggs was recorded during the high patent period (30–70 dpi).

During the present study (2006–2010) a comparable and consistent proportion of dogs from the control areas were infected with *E. intermedius* (between 2.7 and 5.2%), documenting the regular transmission of the parasite in the investigated area. Meanwhile, in the areas with anthelmintic intervention, infections with *E. intermedius* decreased significantly after two years of intervention when compared to dogs in the control areas. The fact that infection was still prevalent in dogs 2 months after the treatment in 2007 is indicative of still existing transmission of *E. intermedius* on farms. The decrease in dog infection was followed by a significant decrease of CE in fatteners and sows in 2008 as compared to the pigs from control areas. During the time of the study, fattener pigs were slaughtered at up to 9 months of age while sows were reared for 2–4 years in traditional backyard farming systems in Lithuania. As sows are more likely to be exposed to the infection with *E. intermedius* over their lifetime and harbour higher numbers of fertile metacestodes, they may play a significant role in the transmission of *E. intermedius*. While CE infection in fatteners represents a recent transmission of the parasite, the infection in sows with partially older cysts may be a result of older infections. However, no published information on the importance of sows as compared to fatteners in the transmission of *E. intermedius* is available. To estimate the evolution over time of metacestodes detected in fatteners and sows, all cysts isolated from pigs were categorised into three groups (developing, fertile and degenerating). However, the real age especially of the degenerated metacestodes mainly found in sows could not be determined. The fact that more sows (617) were slaughtered in the control area as compared with the intervention area (313) is not indicative for a higher infection pressure in the control area. Furthermore, the number of slaughtered sows does not represent the number of growing sows in the respective areas.

The higher number of dogs in the control areas (540) as compared to the treatment areas (413) could have contributed to an increased environmental contamination with taeniid eggs and also an increase of the transmission of *E. intermedius* in the control areas. However, the number of dogs in both groups was rather proportional to the number of homesteads in the villages. Additionally, the average number of dogs was even lower in the control areas (1.4 dogs/homestead) as compared to those of the treatment areas (1.5 dogs/homestead) as estimated at the beginning of the study (December 2006). As the prevalences of *E. intermedius*, *Taenia* spp. and *E. multilocularis* in dogs and the incidences of CE in pigs from the control and treatment areas were comparable in 2006 and 2007, the intensity of infection must have been similar in the two areas at beginning of the study.

The surprising result of this study was the significant decrease of new infections in pigs represented by the number of developing cysts in fatteners and sows already from the second year of the study. Additionally, the incidence of new CE cases in pigs was significantly (*P* < 0.05) lower during the period of 2008–2010 in the areas with anthelmintic intervention as compared to those in the control areas. The efficacy of anthelmintic intervention was also reflected by the reduced (P < 0.05) intensity of cysts in infected pigs in the period of 2008–2010 as compared to those of pigs from control areas.

To the best of our knowledge, this is the first study demonstrating a successful pilot anthelmintic control intervention against *E. intermedius*. The high and fast effect of dog anthelmintic treatment presumably can be attributed to the farm-based epidemiological situation in Lithuania which allowed reaching nearly all dogs on the farms of the control area. Furthermore, the high efficacy of the anthelmintic treatment in the present study was also a consequence of the commitment of the team (Lithuanian University of Health Sciences) which included only people during the whole study period and also due to the easy accessibility of the farms. In contrast, the application of regular supervised praziquantel dosing in a high proportion of dogs has proven to be extremely challenging in endemic areas of South America (Larrieu and Zanini, 2012) and even more pronounced in nomadic or semi-nomadic pastoral communities in Africa (Macpherson, 1995), China (Heath et al., 2006) and Tibet (Huang et al., 2008).

Since praziquantel is effective against other cestodes, the presence of *Taenia* spp. and *E*. *multilocularis* was also investigated in this study. It is well known that *T. hydatigena* persists longer than *E. granulosus* when control measures are applied since it has a higher biotic potential and, therefore, only a few infected dogs not included in the control program can maintain a high infection pressure (Gemmell and Lawson, 1995). In the present study, the excretion of *Taenia* spp. eggs in the treated dogs did not persist longer than those of *E. intermedius*. The presence of CE and *T. hydatigena* cysticercosis (often detected but not quantified in this study) in pigs and also in restrained dogs rely on the old tradition of pig slaughtering on-site and deliberate feeding of dogs with organs. This fact further shows that free access of dogs to carcasses is of minor significance and not relevant for restrained dogs. No significant difference in the prevalence of *E. intermedius* was detected in restrained and unrestrained dogs suggesting that all dogs had comparable access to infected offal.

In earlier study it was reported that majority of village dogs (82.4%) never received anthelmintic treatment and 42.1% of the treated dogs were treated irregularly, often only as puppies (Bružinskaitė et al., 2009). A result from present study supports earlier findings and shows that only 28% of dogs received an anthelmintic treatment at least once before the study. Low deworming frequency of dogs together with comparable access to infected offal suggests that all, restrained, non-restrained and indoor dogs, had comparable contribution in the transmission of *E. intermedius* on the homestead farms.

In the present study, the prevalences of *E. multilocularis* (0.3–1.1%) in the control dogs were comparable to those documented earlier in dogs from Lithuanian villages (0.8%; 2/240) (Bružinskaitė et al., 2009) and similar to prevalences all over Europe (Hegglin and Deplazes, 2013). This data together with genetically confirmed *E. multilocularis* lesions in pigs (0.5%; 3/685) by Bružinskaitė et al. (2009) indicate a persistence of the environmental contamination on the farms. As expected, higher prevalence of *E. multilocularis* was observed in unrestrained dogs with free access to voles, a risk factor described in other European studies (Hegglin and Deplazes, 2013). However, due to the low prevalence, the effect of the regular treatment was more pronounced for *E. intermedius* and *Taenia* spp. infections.

At the time of the intervention, 62.2% of the pigs that were reared in the small family farms were slaughtered in the cold season (Marcinkutė et al., 2015), usually without veterinary inspection of carcasses. The effect of the anthelmintic treatment was reflected in the reduction of CE in pigs, thereby supporting the effectiveness of seasonal treatment during the most intensive slaughtering period. Furthermore, in the areas with anthelmintic intervention, developing metacestodes were only detected during the first (2007; 3.2%) and the second (2008; 1.9%) year of study in fatteners and during the first year (2007; 8.1%) in sows. Later on, the incidence of developing and fertile metacestodes decreased significantly in fatteners and sows reflecting a reduced transmission of *E. intermedius* in villages with anthelmintic intervention.

Interestingly, our evaluation of the sustainability of the intervention 7 years after the end of intervention period (December 2010) revealed a low prevalence of *E. intermedius* and *Taenia* spp. in dogs in both the control and treatment areas. In the last two decades, the population of pigs has decreased in Lithuania from 1.01 million in 2002 to 0.61 million in 2017. At the same time, the proportion of pigs reared on small family farms to the total pig population dropped from 56.7% in 2002 (Statistics, 2002) to 25.2% in 2017 (Statistics, 2018). Additionally, since 2014, various restrictions for pig farming were implemented in Lithuania due to the spread of African swine fever. These restrictions focused especially on the small backyard farms in risk areas primarily along the border in the southeast and later across most of the territory of Lithuania. Together with the general reduction of the dog population, this probably contributed to a lower pressure of infection of *E. intermedius* in the areas investigated and is a likely reason for the low prevalence of all taeniid eggs in dogs from control and treatment areas in 2017. Our pilot control study has shown the feasibility and efficacy of frequent dosing of dogs with praziquantel formulated as bait. Although the reduction of the prevalence in dogs was high, no speculations towards an elimination of *E. intermedius* in the control area or its impact on the transmission for humans is justified based on the small area investigated. Effects on the incidence of CE in humans can only be expected after years of large-scale control of the parasite transmission (Craig et al., 2017). Furthermore, elimination of CE has been documented to take many years, even on insular situations without reintroduction of the parasite. However, the actual epidemiological situation of CE in Lithuania, with strongly reduced transmission of the parasite in some part of the country, represents a unique opportunity for the establishment of a national control strategy against CE. One of the key messages from this study should be that the strategic deworming of dogs during home slaughter season could be more cost effective than blanket prophylactic recommendations such as bimonthly, or even monthly (Craig et al., 2017) deworming year-round. However, in such circumstances all dogs, not only free-roaming dogs, should be targeted given the finding that chained, indoor and penned dogs had similar prevalence of *E. intermedius* as compared to those of unrestrained dogs.

## Financial support

The study was supported by Bayer Animal Health GmbH, Leverkusen, Germany.
